# CEBPA Overexpression Enhances β-Cell Proliferation and Survival

**DOI:** 10.3390/biology13020110

**Published:** 2024-02-09

**Authors:** Peter N. Ellsworth, Jacob A. Herring, Aaron H. Leifer, Jason D. Ray, Weston S. Elison, Peter Daniel Poulson, Jacqueline E. Crabtree, Pam M. Van Ry, Jeffery S. Tessem

**Affiliations:** 1 Department of Nutrition, Dietetics and Food Science, Brigham Young University, Provo, UT 84602, USA; pellswor@uci.edu (P.N.E.); jherrin3@student.byu.edu (J.A.H.); aaronleifer@gmail.com (A.H.L.); welison@ucsd.edu (W.S.E.); jcrabtr2@student.byu.edu (J.E.C.); 2 Department of Microbiology and Molecular Biology, Brigham Young University, Provo, UT 84602, USA; 3 Department of Chemistry and Biochemistry, Brigham Young University, Provo, UT 84602, USA; poulson.pd@gmail.com (P.D.P.); pvanry@byu.edu (P.M.V.R.)

**Keywords:** Nkx6.1, CEBPA, Nr4a3, ER Stress, Ire1a, beta cell, UPR, cell death, cell proliferation

## Abstract

**Simple Summary:**

The objective of this study was to define the role of the transcription factor CEBPA on pancreatic beta cell function, replication, and survival. We have previously shown that Nkx6.1 induces CEBPA expression, and that this correlates with increased beta cell proliferation, insulin secretion, and cell survival. We show that CEBPA can induce beta cell proliferation, but of greater interest, it is able to protect beta cells from glucolipotoxic damage that causes endoplasmic reticulum stress that results in cell death. Our findings suggest that modulation of CEBPA activity could be used as a treatment to protect and expand a patient’s beta cells.

**Abstract:**

A commonality between type 1 and type 2 diabetes is the decline in functional β-cell mass. The transcription factor Nkx6.1 regulates β-cell development and is integral for proper β-cell function. We have previously demonstrated that Nkx6.1 depends on c-Fos mediated upregulation and the nuclear hormone receptors Nr4a1 and Nr4a3 to increase β-cell insulin secretion, survival, and replication. Here, we demonstrate that Nkx6.1 overexpression results in upregulation of the bZip transcription factor CEBPA and that CEBPA expression is independent of c-Fos regulation. In turn, CEBPA overexpression is sufficient to enhance INS-1 832/13 β-cell and primary rat islet proliferation. CEBPA overexpression also increases the survival of β-cells treated with thapsigargin. We demonstrate that increased survival in response to ER stress corresponds with changes in expression of various genes involved in the unfolded protein response, including decreased Ire1a expression. These data show that CEBPA is sufficient to enhance functional β-cell mass by increasing β-cell proliferation and modulating the unfolded protein response.

## 1. Introduction

Decreased functional β-cell mass is characteristic of type 1 and type 2 diabetes. Functional β-cell mass includes insulin secretion rates, β-cell proliferation, and β-cell survival [1]. Therefore, increasing functional β-cell mass could be leveraged to cure both forms of diabetes. While β-cells are generally considered quiescent after adolescence, studies have shown β-cell proliferation in pregnant or obese rodents and humans [2,3,4], indicating that the molecular pathways that control β-cell proliferation are still intact and can be reactivated under proper conditions. We have previously shown that the β-cell transcription factor Nkx6.1 is sufficient to enhance functional β-cell mass [5], and partially functions through c-Fos [6]. Additionally, we have shown the Nkx6.1 dependence on the nuclear hormone receptors Nr4a1 and Nr4a3 to increase β-cell proliferation and survival [7]. Further characterization of the Nkx6.1 pathway may reveal downstream therapeutic targets that can be leveraged to increase functional β-cell mass.

CEBPA is a bZip transcription factor belonging to the CCAAT/enhancer binding family. CEBP homodimerizes bind and regulate target DNA [8]. CEBP is highly expressed in the healthy human pancreas [9]. The CEBP family plays key roles in the homeostasis of proliferating and terminally differentiated cells [10,11]. In various cell types, the CEBP family regulates cellular proliferation and protection against apoptosis [10,12,13]. Furthermore, CEBPA functions as a transcriptional activator or repressor in a context-dependent manner [13]. While the function of CEBPA has been explored in other tissues, its role in the pancreatic β-cell has not been described. We have previously shown that Nkx6.1 induces CEBPA expression in primary rat islets within the first 24 h of overexpression [6]. Given that CEBPA is an early-induced Nkx6.1 target gene, we set out to investigate whether CEBPA is involved in increasing functional β-cell mass.

The endoplasmic reticulum (ER) is central to proper protein folding and maturation. Under diabetic conditions, β-cells often experience conditions that trigger ER stress, such as hyperglycemia and hyperlipidemia. Hyperglycemia can result in a dramatic increase in insulin biosynthesis [14]. Research suggests that enhanced insulin production could increase unfolded proteins in the ER, leading to augmented ER stress and activation of the unfolded protein response [15]. ER stress is sensed through three ER proteins: Inositol Requiring 1 (Ire1a or ERN1), PKR-like ER kinase (PERK), and Activating Transcription Factor 6 (ATF6) [16]. These proteins trigger a complex pathway that alters cellular function to aid the β-cell in maintaining cellular homeostasis [17,18,19].

We show that CEBPA is induced by Nkx6.1 overexpression in INS-1 832/13 β-cells and primary rat islets. Using INS-1 832/13 β-cells and primary rat islets, we show that CEBPA overexpression is sufficient to induce β-cell proliferation. We demonstrate that CEBPA overexpression upregulates Nr4a3 levels, which may induce the Nr4a-mediated proliferation pathway. Furthermore, CEBPA overexpression is sufficient to protect β-cells from thapsigargin and glucolipotoxicity induced cell death, corresponding with decreased Ire1a expression and decreased activation of Caspase 3. These data demonstrate that CEBPA can enhance functional β-cell mass through inducing proliferation and increasing cell survival.

## 2. Materials and Methods

### 2.1. Cell Culture

INS-1 832/13 rat insulinoma β-cells were cultured as previously demonstrated [20]. Briefly, the INS-1 832/13 rat β-cell line was grown in RPMI complete 1640 medium supplemented with L-glutamine, 11.2 mM glucose, 50 μ/mL streptomycin, 10% fetal bovine serum, 50 U/mL penicillin 10 mM HEPES, and INS-1 supplement [5,6,7,20,21].

### 2.2. Islet Isolation and Culture

Wistar rat breeding pairs were obtained from Harlan and fed a standard chow diet (Teklad 7001; Harlan, KY, USA). Pups were weaned at 3 weeks of age. Male rats were fed ad libitum and kept on a 12 h dark–light cycle. Pancreatic islets were isolated from 5-week-old male rats as previously described [21,22]. Primary rat islets were cultured in islet media consisting of RPMI 1640 as previously described, with 1% fungizone antimycotic (Life Technologies, Carlsbad, CA, USA). All animal studies were approved and performed in accordance with Brigham Young University’s IACUC guidelines (IACUC protocol number 22-1118). Procedures for isolating, culture and transducing islets were previously described [7,21,22].

### 2.3. Adenoviral Cloning and Preparation

Recombinant AdCMV-CEBPA, AdCMV-Nkx6.1, AdCMV-c-Fos, AdCMV-LacZ, and AdCMV-GFP were generated and purified as previously described [6,7]. For all experiments, cells and islets were transduced with a starting concentration of ~2 × 10^7^ IFU/mL adenovirus (a multiplicity of infection of ~150). For experiments, INS-1 832/13 β-cells were transduced with AdCMV-CEBPA and AdCMV-c-Fos, and serial dilutions were completed to measure the effects of CEBPA expression at lower MOIs. Therefore, when a range of CEBPA or c-Fos expression is presented, the cells were transduced with the respective adenovirus at MOIs of 150, 75, 37.5, and 18.25 [5,6,7,21].

### 2.4. [methyl-^3^H]-Thymidine Incorporation

Proliferation rates in INS-1 832/13 β-cells and primary rat islets were measured by [methyl-^3^H]-thymidine incorporation following previously described methods [7,21,22]. Briefly, for INS-1 832/13 β-cells, ^3^H-thymidine was added to cells for 15 min and were processed. Islets were treated for 24 h with ^3^H-thymidine, harvested, and processed as previously described. ^3^H-thymidine incorporation was normalized to total cellular protein concentration as determined by bicinchoninic acid assay [5,7,22]. Data are presented relative to no treatment conditions (NT) for either cells or islets.

### 2.5. Cell Viability Assays

INS-1 832/13 β-cells were untreated (hereafter called No virus-NV), or treated with AdCMV-GFP, or AdCMV-CEPBA for 48 h. Afterwards, cells were treated with various apoptotic conditions: either etoposide (9.0 μM), camptothecin (2.0 μM), thapsigargin (0.3 μM) or glucolipotoxicity (GLT, 25 mM Glucose and 0.5 mM Palmitate) for 18 h [21]. Cellular viability was determined using Alamar Blue assays following the manufacturer’s instructions [21].

### 2.6. EdU Incorporation

Islets from rats were treated with EdU (Invitrogen, Waltham, MA, USA) following the manufacturer’s protocols. Islets were dispersed and stained with DAPI and insulin (Fitzgerald, 20IP35 1:7500) as previously described [7,23]. Five sections for each condition were analyzed for EdU signals at 40× and 100× using IMAGEJ software (Version 2.15.0-National Institutes of Health, Bethesda, MD, USA) [23].

### 2.7. Glucose-Stimulated Insulin Secretion

INS-1 832/13 cells or rat islets were used to perform glucose-stimulated insulin secretion (GSIS) assays as previously described [22,24]. Briefly, confluent INS-1 832/13 β-cells were washed with secretion assay buff (SAB) and incubated for 2 h (1.2 mM KH_2_PO_4_, 20 mM HEPES, 114 mM NaCl, 1.16 mM MgSO_4_, 0.2% BSA, 2.5 mM CaCl_2_, and 4.7 mM KCl, pH 7.2) supplemented with 2.5 mM glucose. Following a 2 h wash, triplicate samples were incubated in SAB containing 2.5 mM glucose for 1 h (basal), then for 1 h in SAB with 12.0 mM glucose (stimulatory). Buffers were collected following 1 h incubations. To quantify total insulin content, samples were lysed in RIPA buffer with protease inhibitors (Life Technologies) following stimulatory treatment. Insulin was measured in SAB using an insulin ELISA kit and normalized to total protein content (using BSA assay), as previously described [25].

### 2.8. qPCR

RNA was isolated using TriReagent (Life Technologies) and cDNA was reverse transcribed using the High-Capacity cDNA Reverse Transcription kit (Life Technologies), per the manufacturer’s instructions. cDNA was analyzed using quantitative PCR on the Life Technologies One Step Plus Sequence Detection System and Software (Software version 2.3, Life Technologies). Primers for the following genes were designed for SybrGreen assay and are available upon request: CEBPA, Nr4a3, Nr4a1 VGF, c-Fos, Eif2ak3, Atf6, Eif2a, Chaf1a, Ddit3, Ire1a, Hsf1, Xbp1, Birc5, and PPIA (IDT DNA).

### 2.9. Immunoblot Analysis

Samples for analysis were washed in PBS and resuspended in RIPA buffer, and then sonicated. A quantity of 30 μg of sample was run on a 10% gel as quantified by BCA. Gel transfer, probing, and visualization were completed as previously described [7]. Following transfer, immunoblotting was completed with anti-tubulin (10094-1-AP, Proteintech, Rosemont, IL, USA, 1:2500), anti-Nr4a1 (12235-1-AP, Proteintech, Rosemont, IL, USA, 1:1000), anti-c-Fos (GTX129846, GeneTex, Irvine, CA, USA, 1:2000), anti-CEBPA (2295S, Cell Signaling Technology, Danvers MA, USA, 1:1000), anti-Ire1a (3294S, Cell Signaling Technology, Danvers, MA, USA, 1:1000), anti-VGF (SAB2102676, Sigma, St. Louis, MO, USA, 1:1000), and anti-Nr4a3 (55405-1-AP, Proteintech, Rosemont, IL, USA, 1:1000) antibodies. Images were taken using a Licor Odyssey CLx and analyzed using Image Studio.

### 2.10. Flow Cytometry

INS-1 832/13 β-cells were trypsinized from the growth plate and stained per the manufacturer’s recommendation using a CellEvent Caspase-3/7 Green Flow Cytometry Assay Kit to test Caspase-3/7 activity (Invitrogen, Cat. No. C10427) [26]. After staining, data were acquired using an Attune NxT cytometer (Invitrogen). Data were analyzed using FlowJo (BD Life Sciences, Franklin Lakes, NJ, USA, version 10.8.1).

### 2.11. Statistical Analysis

All results are expressed as mean ± SD. Data were analyzed using a two-tailed Student *t*-test, one-way ANOVA, or two-way ANOVA where appropriate using GraphPad Prism (GraphPad Software, La Jolla, CA, USA, version 10.0.0 (131)). Statistical significance was defined as *p* < 0.05.

## 3. Results

### 3.1. Nkx6.1 Overexpression Induces CEBPA Expression

**Figure 1:**
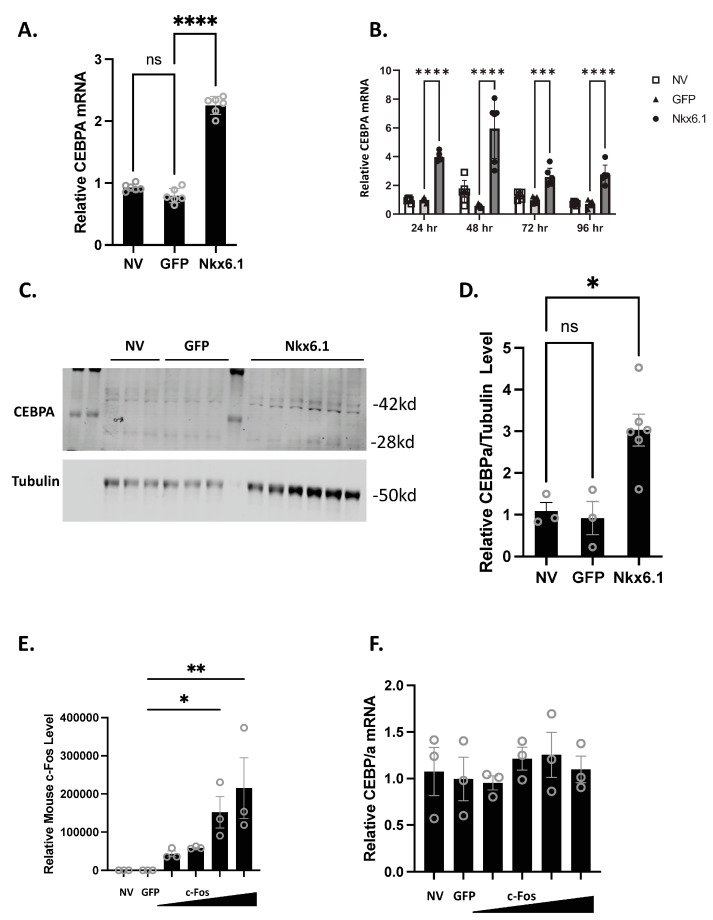
**Nkx6.1 overexpression induces CEBPA expression.** (**A**) INS-1 832/13 β-cells or (**B**) Primary rat islets were transduced with AdCMV-GFP or AdCMV-Nkx6.1 at an MOI of ~150, and induction of CEBPA mRNA levels and protein levels were measured (**C**,**D**). (**E**) Transduction of INS-1 832/13 β-cells with AdCMV-GFP at an MOI of ~150 or AdCMV-c-Fos at an MOI of ~150 and descending by serial dilutions (i.e., 150, 75, 37.5, and 18.8 MOI) expressing mouse c-Fos increases c-Fos levels, but (**F**) fails to induce CEBPA levels. n = 3–6. NV represents “No Virus” and GFP represents AdCMV-GFP. AdCMV-GFP was used at an MOI of ~150. Open circles represent data points. ns = non-significant. * *p* < 0.05, ** *p* < 0.01, *** *p* < 0.001, and **** *p* < 0.0001.

Overexpression of Nkx6.1 is sufficient to induce β-cell proliferation. Nkx6.1-mediated proliferation, however, does not occur until 48 h post-overexpression, suggesting that early targets are necessary for permitting proliferation to occur [7]. We have previously shown that CEBPA is induced as early as 24 h post-Nkx6.1 overexpression [6]. We measured CEBPA in primary rat islets and the INS-1 832/13 β-cell line after transduction with Nkx6.1-expressing adenovirus. We observed that CEBPA was induced 2-fold in INS-1 832/13 β-cells 48 h after transduction (Figure 1A) and between 2- and 6-fold beginning as early as 24 h after adenoviral transduction in primary rat islets (Figure 1B). This corresponds with increased CEBPA protein levels in response to Nkx6.1 overexpression in INS-1 832/13 β-cells (Figure 1C,D). We have previously shown that many of the Nkx6.1 target genes are upregulated through the early Nkx6.1 target gene c-Fos [6]. We sought to determine if c-Fos was sufficient to induce CEBPA expression. INS-1 832/13 β-cells transduced with AdCMV-c-Fos demonstrate a strong induction of mouse c-Fos (Figure 1E); however, no induction of CEBPA was observed (Figure 1F). These data demonstrate that Nkx6.1 overexpression is sufficient to induce CEBPA expression in INS-1 832/13 β-cells and primary islets, and that CEBPA induction is independent of c-Fos.

### 3.2. CEBPA Overexpression Induces Expression of Nr4a3

**Figure 2:**
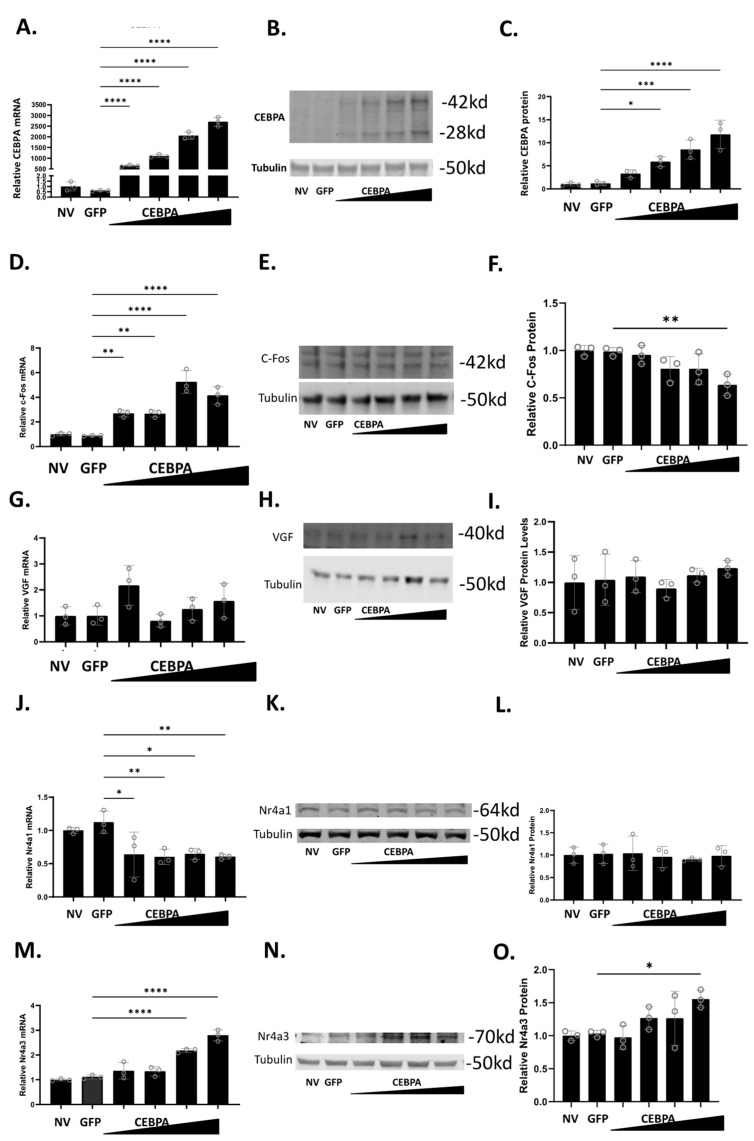
**CEBPA overexpression increases protein level of Nr4a3.** INS-1 832/13 β-cells were untreated or transduced with AdCMV-GFP at an MOI of ~150 or AdCMV-CEBPA at an MOI of ~150 and descending by serial dilutions (i.e., 150, 75, 37.5, 18.8). Following a 48 h incubation, mRNA and protein levels were measured for CEBPA (**A**–**C**), c-Fos (**D**–**F**), VGF (**G**–**I**), Nr4a1 (**J**–**L**), and Nr4a3 (**M**–**O**). Levels of mRNA are presented in (**A**,**D**,**G**,**J**,**M**). Representative Western blots are presented in (**B**,**E**,**H**,**K**,**N**). Quantification for the Western blots are presented in (**C**,**F**,**I**,**L**,**O**). n = 3. NV represents “No Virus”. * *p* < 0.05, ** *p* < 0.01, *** *p* < 0.001, and **** *p* < 0.0001.

To study the effect of CEBPA expression in β-cells, we used a CEBPA overexpression adenovirus, and demonstrated that it is sufficient to increase CEBPA mRNA (Figure 2A) and protein levels (Figure 2B,C). Given that CEBPA can produce two isoforms (p42 and p30) from the same cDNA, both bands were quantified together [27]. We have previously shown that Nkx6.1 induces expression of c-Fos, which induces expression of Nr4a1, Nr4a3, and VGF [6]. The induction of these genes is sufficient to enhance β-cell proliferation, insulin secretion, and survival [6,20]. Therefore, we sought to determine if CEBPA overexpression induces the expression of c-Fos, VGF, Nr4a1, and Nr4a3. CEBPA overexpression increases c-Fos mRNA but fails to increase c-Fos protein (Figure 2D–F). CEBPA overexpression does not change VGF mRNA or protein level (Figure 2G–I). CEBPA decreases Nr4a1 mRNA level but does not change the protein level (Figure 2J–L). Finally, CEBPA induces the expression of Nr4a3 mRNA and protein levels (Figure 2M–O). These data demonstrate that CEBPA overexpression is sufficient to induce Nr4a3 expression but does not increase protein levels of the other Nkx6.1 target genes c-Fos, VGF, or Nr4a1.

### 3.3. CEBPA Overexpression Does Not Alter Glucose-Stimulated Insulin Secretion or Insulin Content

**Figure 3:**
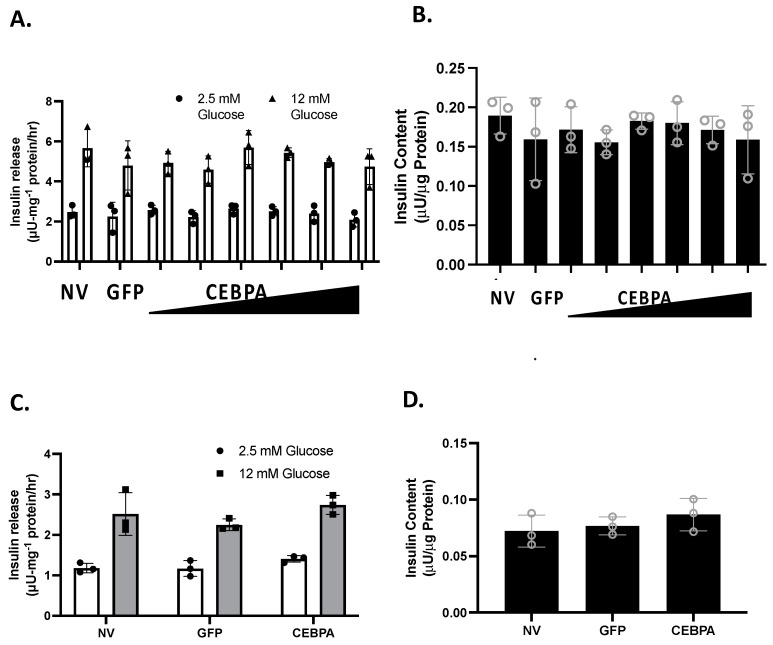
**CEBPA overexpression does not alter glucose stimulated insulin secretion or insulin content.** INS-1 832/13 β-cells were transduced with AdCMV-GFP at an MOI of ~150 or AdCMV-CEBPA at an MOI of ~150 and descending by serial dilutions (i.e., 150, 75, 37.5, and 18.8 MOI). 48 h after adenoviral transduction (**A**) Glucose stimulated insulin secretion was measured in response to 2.5 mM or 12 mM Glucose stimulation, and (**B**) total cellular insulin content was measured. Similarly, (**C**) GSIS was measured at 2.5 and 12 mM glucose in primary rat islets following 48 h of CEBPA overexpression and (**D**) total islet insulin content was measured. n = 3. NV represents “No Virus”.

We have shown that knockdown of the CEBPA target gene Nr4a3 is sufficient to diminish glucose-stimulated insulin secretion (GSIS) [20]. Similarly, the Nkx6.1 target genes c-Fos and VGF are sufficient to enhance GSIS [6,24]. We hypothesized that CEBPA may be able to potentiate GSIS through Nr4a3 induction. We measured GSIS and insulin content in INS-1 832/13 β-cells or primary rat islets that were either untreated or transduced with either AdCMV-CEBPA or AdCMV-GFP. Following 48 h of CEBPA overexpression, we observed that CEBPA did not alter normal levels of GSIS and insulin content levels in INS-1 832/13 β-cells (Figure 3A,B) and primary rat islets (Figure 3C,D). These data demonstrate that CEBPA overexpression does not alter normal GSIS and insulin content.

### 3.4. CEBPA Overexpression Induces β-Cell Proliferation

**Figure 4:**
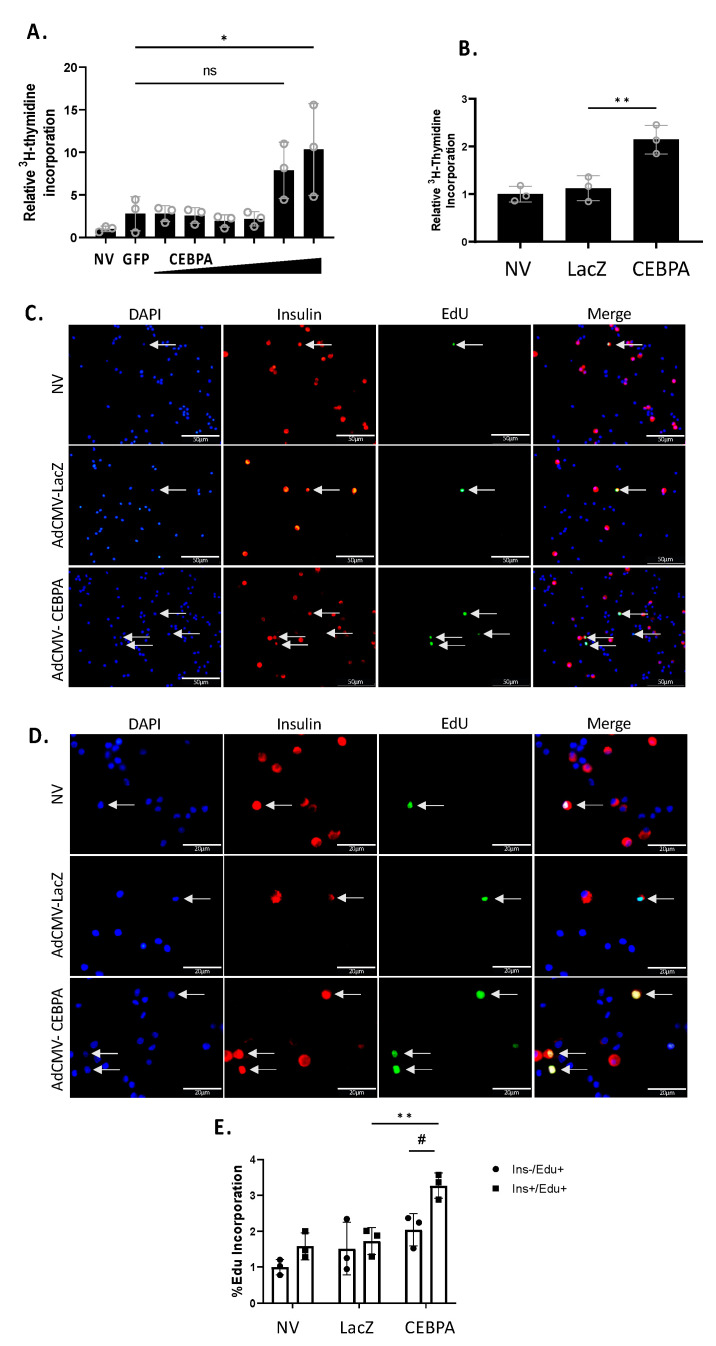
**CEBPA overexpression induces β-cell proliferation.** INS-1 832/13 β-cells were transduced with AdCMV-GFP at an MOI of ~150 or AdCMV-CEBPA at an MOI of ~150 and descending serial dilutions (i.e., 150, 75, 37.5, and 18.8 MOI). Following a 72 h incubation, cells were labelled with ^3^H-thymidine for 15 min and incorporation was measured, indicating DNA replication and proliferation (**A**). Primary rat islets were transduced with AdCMV-LacZ at an MOI of ~150 or AdCMV-CEBPA at an MOI of ~150. Following a 48 h incubation, islets were labelled with ^3^H-thymidine for 24 h and incorporation was measured, indicating DNA replication and proliferation (**B**). Primary rat islets were transduced with AdCMV-LacZ at an MOI of ~150 or AdCMV-CEBPA at an MOI of ~150. Following a 48 h incubation, islets were labelled with EdU for 24 h. Cells were stained for EdU incorporation (green), nuclei (DAPI) and insulin expression (red) to label beta cells and incorporation was measured, indicating DNA replication and proliferation. Select images at 40× (**C**) and 100× (**D**) are presented. Quantification demonstrates a significant increase in β-cell proliferation following CEBPA overexpression and no significant change in non-β-cell proliferation (**E**). ns = not significant, n = 3. * or # *p* < 0.05 and ** *p* < 0.01.

We have previously shown that Nr4a3 can induce β-cell proliferation, and that loss of Nr4a3 impairs β-cell proliferation [7,20]. Given that CEBPA overexpression increases Nr4a3 mRNA and protein levels, we hypothesized that CEBPA would induce proliferation of INS-1 832/13 β-cells and primary rat islets. We measured cellular proliferation using [methyl-^3^H]-thymidine incorporation in INS-1 832/13 β-cells or primary rat islets. Cells or islets were left untreated or transduced with either AdCMV-CEBPA or AdCMV-GFP. INS-1 832/13 β-cells transduced with AdCMV-CEBPA demonstrate a significant induction in proliferation (Figure 4A) as did primary islets (Figure 4B). To validate islet β-cell replication, we measured EdU incorporation after transduction with AdCMV-CEBPA or AdCMV-LacZ (Figure 4C). We demonstrated a significant increase in Insulin^+^ EdU^+^ cells in AdCMV-CEBPA-transduced islets, with no significant change in Insulin^−^ EdU^+^ cells (Figure 4D). These data demonstrate that CEBPA overexpression is sufficient to induce β-cell proliferation in INS-1 832/13 β-cells and primary rat islets.

### 3.5. CEBPA Overexpression Increases Cell Survival in Response to Thapsigargin Treatment and Glucolipotoxicity

**Figure 5:**
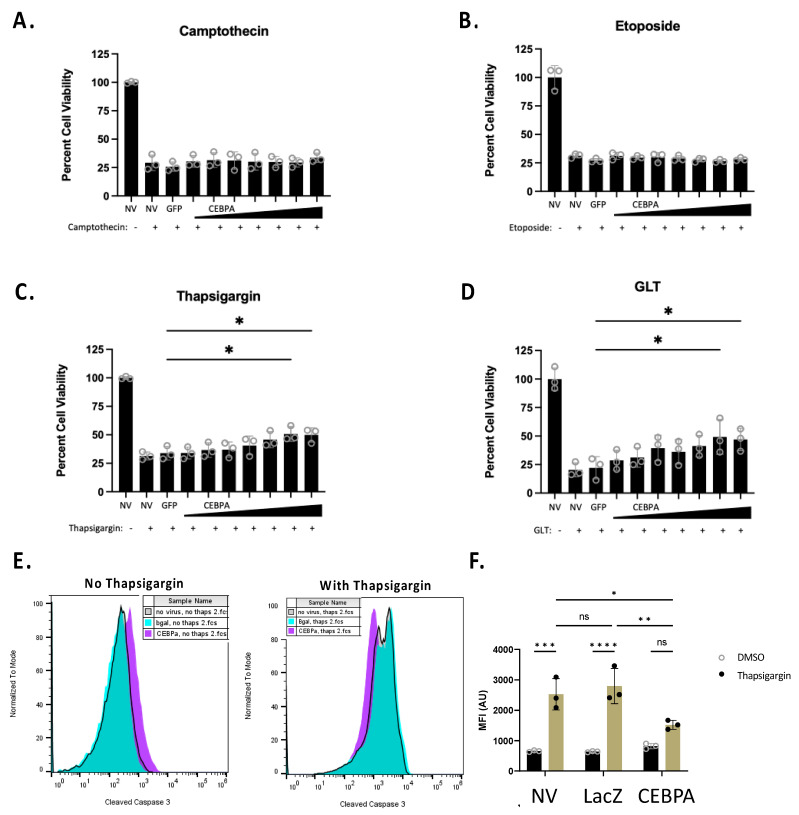
**CEBPA decreases thapsigargin- and glucolipotoxicity-induced cell death.** INS-1 832/13 β-cells were transduced with AdCMV-GFP at an MOI of ~150 or AdCMV-CEBPA at an MOI of ~150 and descending serial dilutions (i.e., 150, 75, 37.5, and 18.8 MOI). Following 48 h of incubation, cells were untreated or cultured with (**A**) camptothecin (2.0 μM), (**B**) etoposide (9.0 μM), (**C**) thapsigargin (0.3 μM), or (**D**) glucolipotoxic conditions (GLT, 25 mM glucose and 0.5 mM palmitate). (**E**) INS-1 832/13 β-cells were transduced with AdCMV-LacZ at an MOI of ~150 or AdCMV-CEBPA at an MOI of ~150, and activated caspase 3 was measured by flow cytometric measurements with and without thapsigargin, (**F**) with quantification to highlight the decrease in activated caspase 3. n = 3. NV represents “No Virus”. ns = not significant, * *p* < 0.05, ** *p* < 0.01, *** *p* < 0.001, and **** *p* < 0.0001.

The Nkx6.1 pathway increases cell survival in response to some β-cell stress inducers [6]. We sought to determine if CEBPA is sufficient to increase β-cell survival in response to various cellular stressors. We transduced INS-1 832/13 β-cells with AdCMV-GFP or serial dilutions of AdCMV-CEBPA, or left untreated. Following 48 h of incubation, we treated cells for 24 h with camptothecin, etoposide, or thapsigargin, or under glucolipotoxic conditions (a total of 72 h after transduction). Camptothecin and etoposide were selected for use, as they are topoisomerase inhibitors and cause general cell death due to DNA damage. Alternatively, thapsigargin and glucolipotoxicity were used, as they impinge on ER stress pathways. We observed no protective effect of CEBPA overexpression on cells treated with the topoisomerase inhibitors camptothecin or etoposide (Figure 5A,B). However, CEBPA overexpression conferred a protective effect on cells treated with thapsigargin or cultured under glucolipotoxic conditions (Figure 5C,D). We measured thapsigargin-mediated caspase 3 activation in the presence of CEBPA overexpression in INS-1 832/13 β-cells. CEBPA overexpression causes a significant decrease in caspase 3 activity in INS-1 832/13 β-cells treated with thapsigargin, as demonstrated by flow cytometry (Figure 5E,F). Given that glucolipotoxicity and thapsigargin induce ER stress, these data suggest that CEBPA overexpression confers protection against ER stress inducers.

### 3.6. CEBPA Modulates Expression of Genes Associated with ER Stress and the Unfolded Protein Response

**Figure 6:**
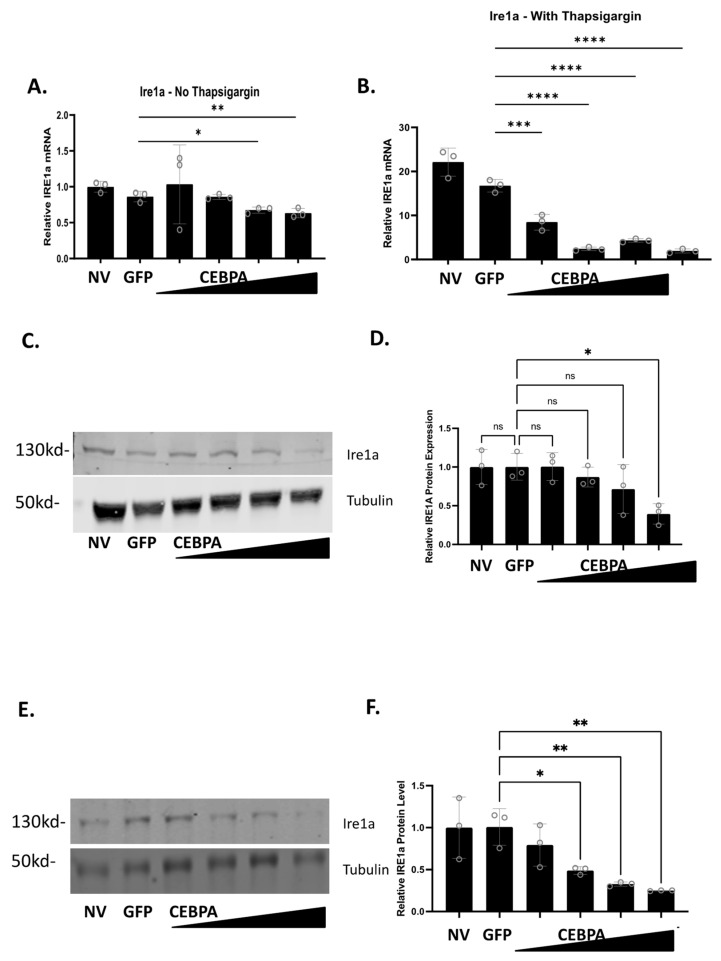
**CEBPA overexpression decreases Ire1a mRNA and protein levels in the presence of thapsigargin**. INS-1 832/13 β-cells were transduced with AdCMV-GFP at an MOI of ~150 or AdCMV-CEBPA at an MOI of ~150 and descending serial dilutions (i.e., 150, 75, 37.5, and 18.8 MOI). Following 48 h of incubation, cells were cultured with or without thapsigargin for 24 h. mRNA levels for (**A**) Ire1a without thapsigargin and (**B**) with thapsigargin was measured. mRNA of Ire1a with thapsigargin is normalized to the no thapsigargin-NV control. Western blot analysis of (**C**) Ire1a without thapsigargin with (**D**) quantitation, and (**E**) Ire1a with thapsigargin with (**F**) quantitation are presented. n = 3. NV represents “No Virus”. ns = not significant, * *p* < 0.05, ** *p* < 0.01, *** *p* < 0.001, **** *p* < 0.0001.

Given the observed phenotype that CEBPA overexpression confers protection to thapsigargin and glucolipotoxicity, both of which induce ER stress and activation of the unfolded protein response (UPR), we sought to determine how CEBPA overexpression affects the expression of genes associated with ER stress and the UPR. INS-1 832/13 β-cells were untreated or transduced with either AdCMV-GFP or AdCMV-CEBPA. After a 48 h incubation, cells were treated with or without thapsigargin for 24 h. CEBPA overexpression without thapsigargin had no change on the mRNA levels of Hsf1, Eif2a, Birc5, Atf4, Ddit3, P4hb, and Chaf1a (Figure S1A–E,G,K). CEBPA overexpression without thapsigargin resulted in increased expression of Xbp1, Eif2ak3 (PERK), and Atf6 (Figure S1F,I,J), and decreased expression of Hspa5 (Figure S1H), and Ire1a (Figure 6A). When CEBPA overexpressing cells were cultured for 24 h with thapsigargin, neither Hspa5 nor Atf6 had any change in expression compared to the controls (Figure S2H,J). Increased expression was observed for Eif2a, Birc5, and Chaf1a with CEBPA overexpression and thapsigargin treatment (Figure S2B,C,K). Significant decreased expression with CEBPA overexpression and thapsigargin treatment was observed for Hsf1, Atf4, Ddit3, Xbp1, P4hb, Eif2ak3 (Figure S2A,D,E–G,I), and Ire1a (Figure 6B).

Our data demonstrate that CEBPA overexpression decreases mRNA expression of Ire1a in the presence and absence of thapsigargin. Therefore, we measured Ire1a protein levels to validate Ire1a changes as a function of CEBPA overexpression. Untreated INS-1 832/13 β-cells, or those transduced with AdCMV-GFP or AdCMV-CEBPA, were treated with or without thapsigargin, and Ire1a protein levels were measured. We observed that Ire1a protein levels decrease as CEBPA levels increase in the absence (Figure 6C,D) or in the presence of thapsigargin (Figure 6E,F). Together, these data suggest that CEBPA mediated protection against UPR induced cell death occurs due to modulation of critical proteins in the ER stress and UPR pathway, including downregulation of Ire1a protein levels which would crucially downregulate a key portion of the UPR. Furthermore, our data demonstrate that CEBPA overexpression is sufficient to decrease Ire1a mRNA and protein, independent of thapsigargin treatment.

## 4. Discussion

Loss of functional β-cell mass is a hallmark of both Type 1 and Type 2 diabetes. Defining the molecular pathways that allow for increased β-cell proliferation, insulin secretion, and cell survival is imperative in designing therapeutic interventions for individuals with diabetes. Here, we demonstrate that the bZip transcription factor CEBPA is sufficient to enhance functional β-cell mass through increasing β-cell proliferation and conferring protection against cellular insults that result in ER stress and activation of the UPR. We demonstrate that CEBPA induces expression of Nr4a3, which we have previously shown is sufficient to induce β-cell proliferation [7] and may be part of the CEBPA-mediated proliferation pathway. We have also shown that CEBPA induces downregulation of Ire1a, which is critical for activation of the UPR and inducing cell death in response to damage such as that caused by thapsigargin or glucolipotoxicity.

In this study, we demonstrate the upregulation of genes such as Nr4a3, and downregulation of others such as Ire1a in response to CEBPA overexpression. While CEBPA has generally been characterized as a transcription activator, our study and others have also profiled its role as a transcription repressor. Studies in adult hematopoietic stem cells demonstrate activation and inhibition of gene expression due to CEBPA [28]. Similar results are shown for CEBPA in a leukemogenesis model, demonstrating both transactivating and repressing activities [13,29]. The CEBPA transcriptome has not yet been defined in β-cells. Given our observed results, this is of future interest.

Our data demonstrate that CEBPA overexpression is sufficient to induce proliferation of primary rat islets and β-cells. Furthermore, our data demonstrate that CEBPA induces expression of Nr4a3, which we have previously shown is sufficient to induce β-cell proliferation [7,20]. While this does not prove that the increase in Nr4a3 leads to the increase in proliferation, it does hint at a potential mechanism for how CEBPA overexpression is inducing proliferation. There are no published reports connecting CEBPA directly to Nr4a3 expression, although signaling pathways that induce Nr4a3 have also been shown to induce CEBPA [30]. Interestingly, Nr4a1 and Nr4a3 have been shown to inhibit CEBPA expression in hematopoietic stem cells, suggesting a potential feedback mechanism to the Nr4a3 induction that we observed in β-cells [31,32]. CEBPA has been shown to play roles in cell cycle entry and proliferation of other tissue types. CEBPA levels are critical for cell cycle gene expression in regenerating liver [33]. CEBPA is necessary for proliferation and differentiation of adipocytes [34]. Other studies have suggested, however, that CEBPA plays a key role in exiting the cell cycle and impeding cellular proliferation [35]. The proliferation observed in our model in comparison to others where proliferation is not observed may reflect the difference between β-cells, which are largely post-mitotic, and other tissues that have higher replicative potential [36].

Our data also demonstrate that CEBPA overexpression protects β-cells from ER stress, which results in activation of the UPR. Our finding is supported by studies that show that CEBPA enables adaptation to REDOX stress in acute myeloid leukemia [13]. Furthermore, activation of the UPR is associated with decreased CEBPA expression, supporting our data that elevated CEBPA may inhibit UPR progression [37]. While our data shows that CEBPA can inhibit the UPR, other studies contradict our findings. Studies in failing human heart samples demonstrate increased expression of UPR genes and CEBPA [38]. While there are conflicting results regarding the role of CEBPA in ER stress and the UPR, our data demonstrate a strong modulation of genes involved in the UPR. The strongest observed change was downregulation of Ire1a mRNA and protein levels in the absence or presence of thapsigargin treatment. Ire1a is the initiating factor in one of the three primary pathways of the UPR. Ire1a activation results in cleavage of Xbp1 mRNA, which was also downregulation in our model [39]. Our study is not the first to demonstrate that Ire1a downregulation can decrease UPR-mediated cell death. Inhibiting Ire1a activity in a mouse model of Kawasaki disease vasculitis was sufficient to decrease cell death [40]. Furthermore, in a human acute myeloid leukemia study, patients that had defective CEBPA double mutants had increased Ire1a expression [41]. Future studies will look at the effect of GLT on Ire1a levels with CEBPA overexpression. In addition to the Ire1a pathway, a strong decrease was seen in the mRNA expression of the Eif2ak3 (PERK) pathway [39]. Eif2ak3 activates Atf4, which induces expression of Ddit3 (CHOP), which enhances UPR-mediated cell death [42]. All three of these genes have downregulated mRNA levels in our study in the presence of CEBPA overexpression and thapsigargin treatment, emphasizing the connection to decreased activation of the different arms of the UPR. Future studies will need to be performed to understand the effects of these changes on β-cell survival. CEBPA has been shown to have an antagonistic action towards Ddit3 activity in hepatocytes [43]. We also see upregulation of Birc5 and Chaf1a mRNA, which play critical roles in inhibiting the UPR [44,45]. Supporting these findings are reports showing that CEBPA knockdown decreases Chaf1a expression, and that patients with acute myeloid leukemia that have CEBPA double mutations have decreased Chaf1a levels [41,46,47]. Additionally, a previous study showed that CEBPA was associated with enhanced cytokine-mediated cell death in a β-cell model [48]. Exposure to cytokines can induce the UPR in a β-cell [49]. Given these data, future studies will be needed to explain why CEBPA does not protect against cytokine mediated cell death.

## 5. Conclusions

Our findings demonstrate the ability of CEBPA to increase functional β-cell mass in terms of enhancing β-cell proliferation and protecting against β-cell death induced by ER stress and UPR activation. Given the strong correlation of type 2 diabetes with hyperglycemia and hyperlipidemia, these findings are particularly useful in determining future therapeutics. Hyperglycemia and hyperlipidemia are associated with β-cell oxidative stress and defects in protein processing. This directly affects that secretory nature of the β-cell. Our finding that CEBPA plays a direct role in controlling expression of genes that impinge on protein folding, ER stress, and the UPR suggests that it may be leveraged as a potential intervention to expand functional β-cell mass for patients with type 2 diabetes.

## Data Availability

The data presented in this study are available in this article.

## Supplementary Materials

The following supporting information can be downloaded at: https://www.mdpi.com/article/10.3390/biology13020110/s1, Figure S1: CEBPA overexpression changes expression levels of genes associated with ER stress and the Unfolded Protein Response when ER stress is not induced with thapsigargin. INS-1 832/13 β-cells were not treated with thapsigargin following adenoviral transduction (AdCMV-GFP or AdCMV-CEBPA) 48 h earlier. mRNA levels for (A) Hsf1, (B) Eif2a, (C) Birc5, (D) Atf4, (E) Ddit3, (F), Xbp1, (G) P4hb, (H) Hspa5, (I) Eif2ak3, (J) Atf6, and (K) Chaf1a were measured and normalized to no thapsigargin-no virus control, with comparisons made to the AdCMV-GFP control. n = 3. MOI approximately 150×. * *p* < 0.05, ** *p* < 0.01. Figure S2: CEBPA overexpression changes expression levels of genes associated with ER stress and the Unfolded Protein Response when ER stress is induced with thapsigargin. INS-1 832/13 β-cells were treated with thapsigargin for 24 h following adenoviral transduction (AdCMV-GFP or AdCMV-CEBPA) 48 h earlier. mRNA levels for (A) Hsf1, (B) Eif2a, (C) Birc5, (D) Atf4, (E) Ddit3, (F), Xbp1, (G) P4hb, (H) Hspa5, (I) Eif2ak3, (J) Atf6, and (K) Chaf1a were measured and normalized to no thapsigargin-no virus control, with comparisons made to the AdCMV-GFP control. n = 3. MOI approximately 150×. * *p* < 0.05, ** *p* < 0.01, *** *p* < 0.001, **** *p* < 0.0001.

## References

[1] Weir G.C., Bonner-Weir S. (2013). Islet beta cell mass in diabetes and how it relates to function, birth, and death. Ann. N. Y. Acad. Sci..

[2] Taylor B.L., Benthuysen J., Sander M. (2015). Postnatal beta-cell proliferation and mass expansion is dependent on the transcription factor Nkx6.1. Diabetes.

[3] Uchida T., Nakamura T., Hashimoto N., Matsuda T., Kotani K., Sakaue H., Kido Y., Hayashi Y., Nakayama K.I., White M.F. (2005). Deletion of Cdkn1b ameliorates hyperglycemia by maintaining compensatory hyperinsulinemia in diabetic mice. Nat. Med..

[4] Hull R.L., Kodama K., Utzschneider K.M., Carr D.B., Prigeon R.L., Kahn S.E. (2005). Dietary-fat-induced obesity in mice results in beta cell hyperplasia but not increased insulin release: Evidence for specificity of impaired beta cell adaptation. Diabetologia.

[5] Schisler J.C., Fueger P.T., Babu D.A., Hohmeier H.E., Tessem J.S., Lu D., Becker T.C., Naziruddin B., Levy M., Mirmira R.G. (2008). Stimulation of human and rat islet beta-cell proliferation with retention of function by the homeodomain transcription factor Nkx6.1. Mol. Cell. Biol..

[6] Ray J.D., Kener K.B., Bitner B.F., Wright B.J., Ballard M.S., Barrett E.J., Hill J.T., Moss L.G., Tessem J.S. (2016). Nkx6.1-mediated insulin secretion and beta-cell proliferation is dependent on upregulation of c-Fos. FEBS Lett..

[7] Tessem J.S., Moss L.G., Chao L.C., Arlotto M., Lu D., Jensen M.V., Stephens S.B., Tontonoz P., Hohmeier H.E., Newgard C.B. (2014). Nkx6.1 regulates islet beta-cell proliferation via Nr4a1 and Nr4a3 nuclear receptors. Proc. Natl. Acad. Sci. USA.

[8] Miller M., Shuman J.D., Sebastian T., Dauter Z., Johnson P.F. (2003). Structural basis for DNA recognition by the basic region leucine zipper transcription factor CCAAT/enhancer-binding protein alpha. J. Biol. Chem..

[9] Antonson P., Xanthopoulos K.G. (1995). Molecular cloning, sequence, and expression patterns of the human gene encoding CCAAT/enhancer binding protein alpha (C/EBP alpha). Biochem. Biophys. Res. Commun..

[10] Marchildon F., Fu D., Lala-Tabbert N., Wiper-Bergeron N. (2016). CCAAT/enhancer binding protein beta protects muscle satellite cells from apoptosis after injury and in cancer cachexia. Cell Death Dis..

[11] Lopez R.G., Garcia-Silva S., Moore S.J., Bereshchenko O., Martinez-Cruz A.B., Ermakova O., Kurz E., Paramio J.M., Nerlov C. (2009). C/EBPalpha and beta couple interfollicular keratinocyte proliferation arrest to commitment and terminal differentiation. Nat. Cell Biol..

[12] Miyagawa S., Iguchi T. (2015). Epithelial estrogen receptor 1 intrinsically mediates squamous differentiation in the mouse vagina. Proc. Natl. Acad. Sci. USA.

[13] Sabatier M., Birsen R., Lauture L., Mouche S., Angelino P., Dehairs J., Goupille L., Boussaid I., Heiblig M., Boet E. (2023). C/EBPalpha Confers Dependence to Fatty Acid Anabolic Pathways and Vulnerability to Lipid Oxidative Stress-Induced Ferroptosis in FLT3-Mutant Leukemia. Cancer Discov..

[14] Goodge K.A., Hutton J.C. (2000). Translational regulation of proinsulin biosynthesis and proinsulin conversion in the pancreatic beta-cell. Semin. Cell Dev. Biol..

[15] Harding H.P., Lackey J.G., Hsu H.C., Zhang Y., Deng J., Xu R.M., Damha M.J., Ron D. (2008). An intact unfolded protein response in Trpt1 knockout mice reveals phylogenic divergence in pathways for RNA ligation. RNA.

[16] Fonseca S.G., Gromada J., Urano F. (2011). Endoplasmic reticulum stress and pancreatic beta-cell death. Trends Endocrinol. Metab..

[17] Takayanagi S., Fukuda R., Takeuchi Y., Tsukada S., Yoshida K. (2013). Gene regulatory network of unfolded protein response genes in endoplasmic reticulum stress. Cell Stress. Chaperones.

[18] Ron D., Walter P. (2007). Signal integration in the endoplasmic reticulum unfolded protein response. Nat. Rev. Mol. Cell Biol..

[19] Kozutsumi Y., Segal M., Normington K., Gething M.J., Sambrook J. (1988). The presence of malfolded proteins in the endoplasmic reticulum signals the induction of glucose-regulated proteins. Nature.

[20] Reynolds M.S., Hancock C.R., Ray J.D., Kener K.B., Draney C., Garland K., Hardman J., Bikman B.T., Tessem J.S. (2016). beta-Cell deletion of Nr4a1 and Nr4a3 nuclear receptors impedes mitochondrial respiration and insulin secretion. Am. J. Physiol. Endocrinol. Metab..

[21] Hobson A., Draney C., Stratford A., Becker T.C., Lu D., Arlotto M., Tessem J.S. (2015). Aurora Kinase A is critical for the Nkx6.1 mediated beta-cell proliferation pathway. Islets.

[22] Fueger P.T., Schisler J.C., Lu D., Babu D.A., Mirmira R.G., Newgard C.B., Hohmeier H.E. (2008). Trefoil factor 3 stimulates human and rodent pancreatic islet beta-cell replication with retention of function. Mol. Endocrinol..

[23] Simonett S.P., Shin S., Herring J.A., Bacher R., Smith L.A., Dong C., Rabaglia M.E., Stapleton D.S., Schueler K.L., Choi J. (2021). Identification of direct transcriptional targets of NFATC2 that promote beta cell proliferation. J. Clin. Investig..

[24] Stephens S.B., Schisler J.C., Hohmeier H.E., An J., Sun A.Y., Pitt G.S., Newgard C.B. (2012). A VGF-derived peptide attenuates development of type 2 diabetes via enhancement of islet beta-cell survival and function. Cell Metab..

[25] Krueger E.S., Beales J.L., Russon K.B., Elison W.S., Davis J.R., Hansen J.M., Neilson A.P., Hansen J.M., Tessem J.S. (2021). Gut Metabolite Trimethylamine N-Oxide Protects INS-1 beta-Cell and Rat Islet Function under Diabetic Glucolipotoxic Conditions. Biomolecules.

[26] Valdoz J.C., Franks N.A., Cribbs C.G., Jacobs D.J., Dodson E.L., Knight C.J., Poulson P.D., Garfield S.R., Johnson B.C., Hemeyer B.M. (2022). Soluble ECM promotes organotypic formation in lung alveolar model. Biomaterials.

[27] Lin F.T., MacDougald O.A., Diehl A.M., Lane M.D. (1993). A 30-kDa alternative translation product of the CCAAT/enhancer binding protein alpha message: Transcriptional activator lacking antimitotic activity. Proc. Natl. Acad. Sci. USA.

[28] Ye M., Zhang H., Amabile G., Yang H., Staber P.B., Zhang P., Levantini E., Alberich-Jorda M., Zhang J., Kawasaki A. (2013). C/EBPa controls acquisition and maintenance of adult haematopoietic stem cell quiescence. Nat. Cell Biol..

[29] Collins C., Wang J., Miao H., Bronstein J., Nawer H., Xu T., Figueroa M., Muntean A.G., Hess J.L. (2014). C/EBPalpha is an essential collaborator in Hoxa9/Meis1-mediated leukemogenesis. Proc. Natl. Acad. Sci. USA.

[30] Myers S.A., Eriksson N., Burow R., Wang S.C., Muscat G.E. (2009). Beta-adrenergic signaling regulates NR4A nuclear receptor and metabolic gene expression in multiple tissues. Mol. Cell Endocrinol..

[31] Freire P.R., Conneely O.M. (2018). NR4A1 and NR4A3 restrict HSC proliferation via reciprocal regulation of C/EBPalpha and inflammatory signaling. Blood.

[32] Mullican S.E., Zhang S., Konopleva M., Ruvolo V., Andreeff M., Milbrandt J., Conneely O.M. (2007). Abrogation of nuclear receptors Nr4a3 and Nr4a1 leads to development of acute myeloid leukemia. Nat. Med..

[33] Jakobsen J.S., Waage J., Rapin N., Bisgaard H.C., Larsen F.S., Porse B.T. (2013). Temporal mapping of CEBPA and CEBPB binding during liver regeneration reveals dynamic occupancy and specific regulatory codes for homeostatic and cell cycle gene batteries. Genome Res..

[34] Lefterova M.I., Zhang Y., Steger D.J., Schupp M., Schug J., Cristancho A., Feng D., Zhuo D., Stoeckert C.J., Liu X.S. (2008). PPARgamma and C/EBP factors orchestrate adipocyte biology via adjacent binding on a genome-wide scale. Genes Dev..

[35] Johnson P.F. (2005). Molecular stop signs: Regulation of cell-cycle arrest by C/EBP transcription factors. J. Cell Sci..

[36] Herring J.A., Elison W.S., Tessem J.S. (2019). Function of Nr4a Orphan Nuclear Receptors in Proliferation, Apoptosis and Fuel Utilization Across Tissues. Cells.

[37] Haefliger S., Klebig C., Schaubitzer K., Schardt J., Timchenko N., Mueller B.U., Pabst T. (2011). Protein disulfide isomerase blocks CEBPA translation and is up-regulated during the unfolded protein response in AML. Blood.

[38] Jensen B.C., Bultman S.J., Holley D., Tang W., de Ridder G., Pizzo S., Bowles D., Willis M.S. (2017). Upregulation of autophagy genes and the unfolded protein response in human heart failure. Int. J. Clin. Exp. Med..

[39] Hetz C. (2012). The unfolded protein response: Controlling cell fate decisions under ER stress and beyond. Nat. Rev. Mol. Cell Biol..

[40] Marek-Iannucci S., Yildirim A.D., Hamid S.M., Ozdemir A.B., Gomez A.C., Kocaturk B., Porritt R.A., Fishbein M.C., Iwawaki T., Noval Rivas M. (2022). Targeting IRE1 endoribonuclease activity alleviates cardiovascular lesions in a murine model of Kawasaki disease vasculitis. JCI Insight.

[41] Taskesen E., Bullinger L., Corbacioglu A., Sanders M.A., Erpelinck C.A., Wouters B.J., van der Poel-van de Luytgaarde S.C., Damm F., Krauter J., Ganser A. (2011). Prognostic impact, concurrent genetic mutations, and gene expression features of AML with CEBPA mutations in a cohort of 1182 cytogenetically normal AML patients: Further evidence for CEBPA double mutant AML as a distinctive disease entity. Blood.

[42] Mihailidou C., Papavassiliou A.G., Kiaris H. (2014). A crosstalk between p21 and UPR-induced transcription factor C/EBP homologous protein (CHOP) linked to type 2 diabetes. Biochimie.

[43] Chikka M.R., McCabe D.D., Tyra H.M., Rutkowski D.T. (2013). C/EBP homologous protein (CHOP) contributes to suppression of metabolic genes during endoplasmic reticulum stress in the liver. J. Biol. Chem..

[44] Gundamaraju R., Vemuri R., Chong W.C., Myers S., Norouzi S., Shastri M.D., Eri R. (2018). Interplay between Endoplasmic Reticular Stress and Survivin in Colonic Epithelial Cells. Cells.

[45] Shen J., Liu X., Zhou M., Liu H., Xu L., Meng X. (2020). CHAF1A overexpression in human retinoblastoma promotes cell proliferation and suppresses apoptosis. J. Balk. Union Oncol..

[46] Nurminen V., Neme A., Seuter S., Carlberg C. (2019). Modulation of vitamin D signaling by the pioneer factor CEBPA. Biochim. Biophys. Acta Gene Regul. Mech..

[47] Warwick T., Schulz M.H., Gunther S., Gilsbach R., Neme A., Carlberg C., Brandes R.P., Seuter S. (2021). A hierarchical regulatory network analysis of the vitamin D induced transcriptome reveals novel regulators and complete VDR dependency in monocytes. Sci. Rep..

[48] Barbagallo D., Condorelli A.G., Piro S., Parrinello N., Floyel T., Ragusa M., Rabuazzo A.M., Storling J., Purrello F., Di Pietro C. (2014). CEBPA exerts a specific and biologically important proapoptotic role in pancreatic beta cells through its downstream network targets. Mol. Biol. Cell.

[49] Cardozo A.K., Ortis F., Storling J., Feng Y.M., Rasschaert J., Tonnesen M., Van Eylen F., Mandrup-Poulsen T., Herchuelz A., Eizirik D.L. (2005). Cytokines downregulate the sarcoendoplasmic reticulum pump Ca^2+^ ATPase 2b and deplete endoplasmic reticulum Ca^2+^, leading to induction of endoplasmic reticulum stress in pancreatic beta-cells. Diabetes.

