# Supplementary material for: CEBPA Overexpression Enhances β-Cell Proliferation and Survival
Source: Biology (Basel). 2024 Feb 9;13(2):110. doi: 10.3390/biology13020110 (PMC10887016; doi:10.3390/biology13020110)
Supplement: Supplementary file 1 [file biology-13-00110-s001.zip › biology-2825623-supplementary.pdf]

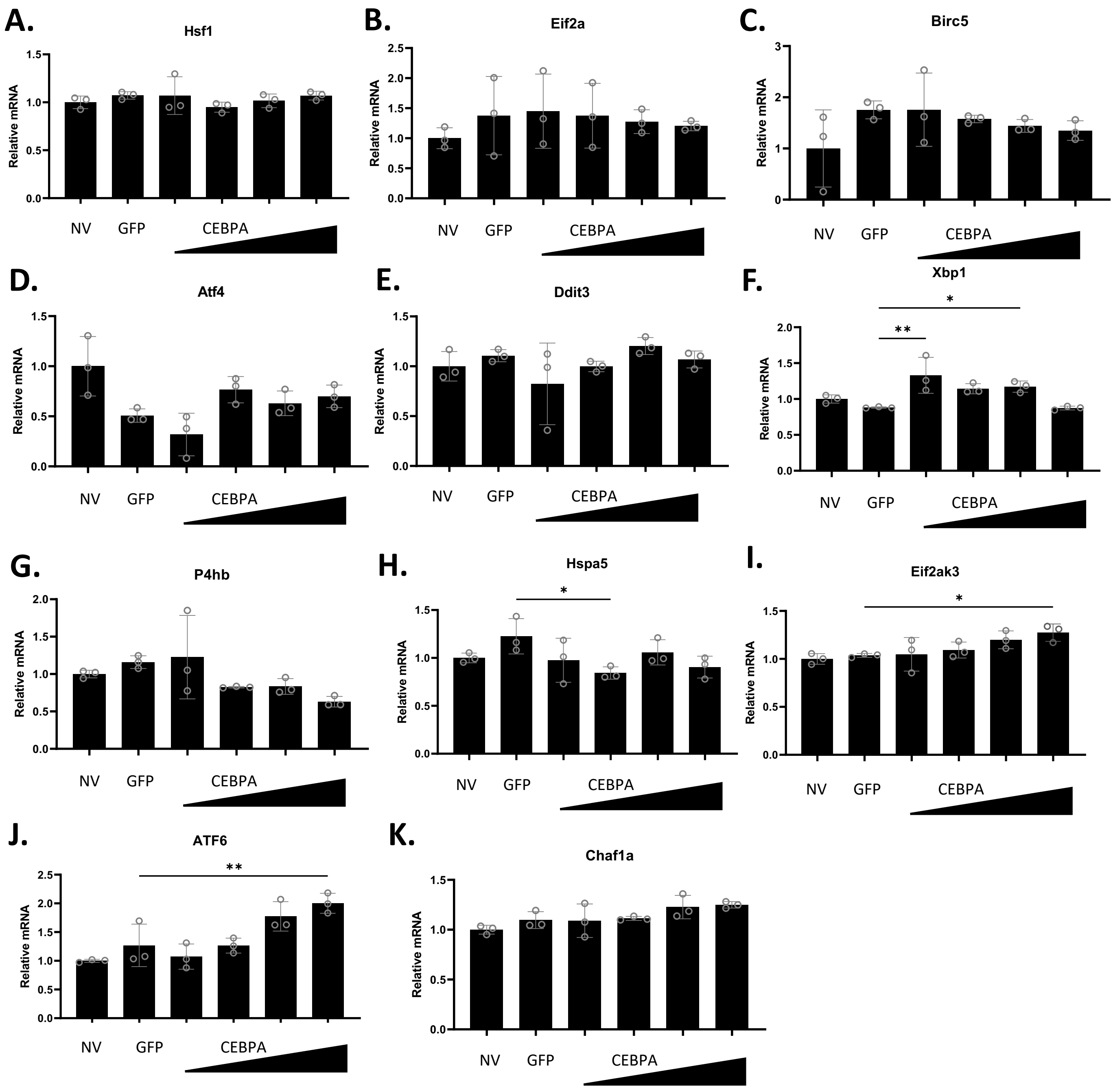

Figure S1: **CEBPA overexpression changes expression levels of genes associated with ER stress and the Unfolded Protein Response when ER stress is not induced with thapsigargin**. INS-1 832/13 β-cells were not treated with thapsigargin following adenoviral transduction (AdCMV-GFP or AdCMV-CEBPA) 48 hours earlier. mRNA levels for (A) Hsf1, (B) Eif2a, (C) Birc5, (D) Atf4, (E) Ddit3, (F), Xbp1, (G) P4hb, (H) Hspa5, (I) Eif2ak3, (J) Atf6, and (K) Chaf1a were measured, with comparisons made to the AdCMV-GFP control. n=3. MOI approximately 150X. * $p < 0.05$, ** $p < 0.01$.

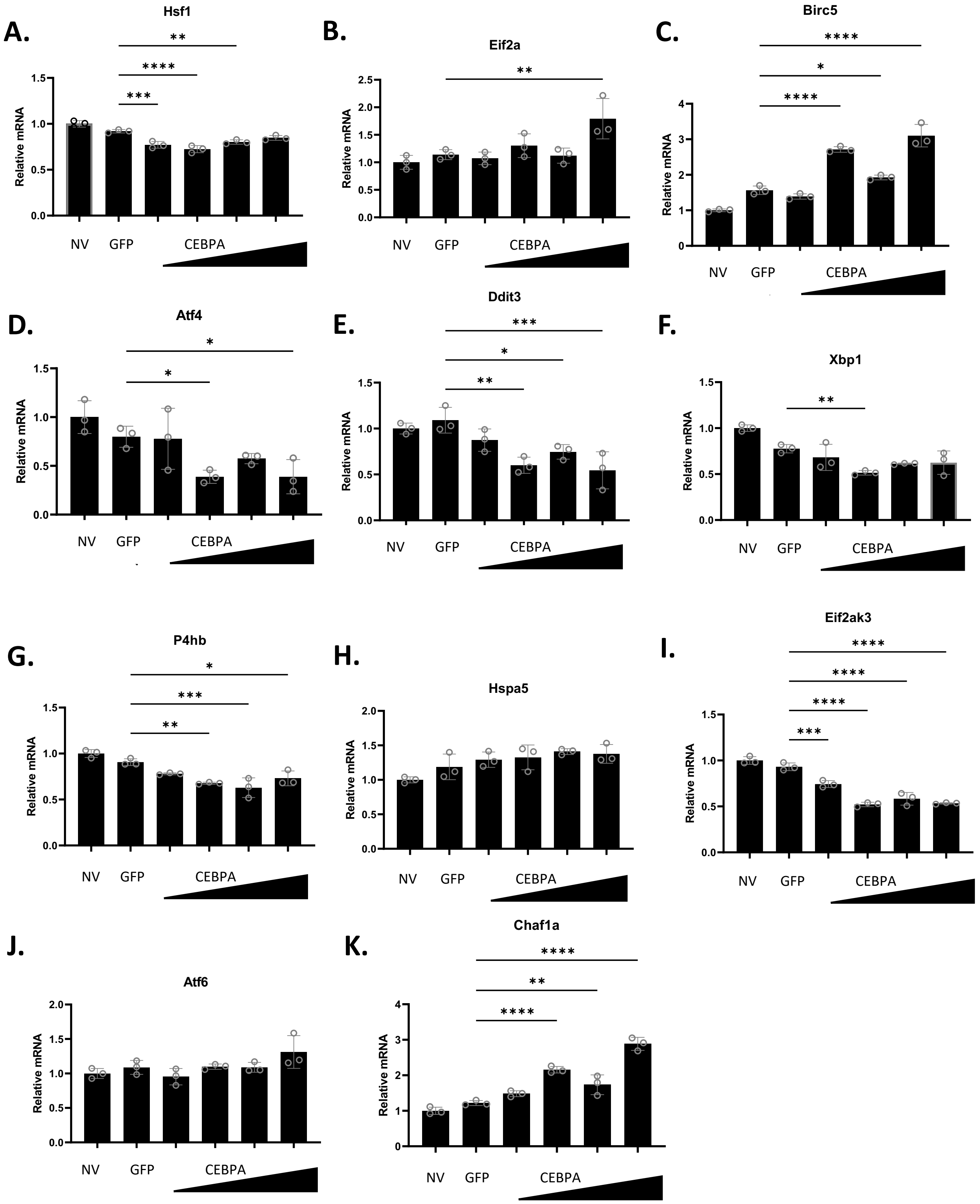

Figure S2: CEBPA overexpression changes expression levels of genes associated with ER stress and the Unfolded Protein Response when ER stress is induced with thapsigargin. INS-1 832/13 β-cells were treated with thapsigargin for 24 hours following adenoviral transduction (AdCMV-GFP or AdCMV-CEBPA) 48 hours earlier. mRNA levels for (A) Hsf1, (B) Eif2a, (C) Birc5, (D) Atf4, (E) Ddit3, (F), Xbp1, (G) P4hb, (H) Hspa5, (I) Eif2ak3, (J) Atf6, and (K) Chaf1a were measured, with comparisons made to the AdCMV-GFP control. n=3. MOI approximately 150X. * p< 0.05, ** p< 0.01, ***p<0.001, ****p<0.0001.
